# Exploring the black box of human reproduction: endometrial organoids and assembloids - generation, implantation modeling, and future clinical perspectives

**DOI:** 10.3389/fcell.2024.1482054

**Published:** 2024-10-23

**Authors:** Mária Kleinová, Ivan Varga, Michaela Čeháková, Martin Valent, Martin Klein

**Affiliations:** ^1^ Institute of Histology and Embryology, Faculty of Medicine, Comenius University in Bratislava, Bratislava, Slovakia; ^2^ Institute of Medical Biology, Genetics and Clinical Genetics, Faculty of Medicine, Comenius University in Bratislava, Bratislava, Slovakia; ^3^ Department of Gynecology and Obstetrics, University Hospital Bratislava – Kramáre Workplace, Bratislava, Slovakia

**Keywords:** endometrial organoids, assembloids, implantation modeling, infertility, 3D modeling

## Abstract

One of the critical processes in human reproduction that is still poorly understood is implantation. The implantation of an early human embryo is considered a significant limitation of successful pregnancy. Therefore, researchers are trying to develop an ideal model of endometrium *in vitro* that can mimic the endometrial micro-environment *in vivo* as much as possible. The ultimate goal of endometrial modeling is to study the molecular interactions at the embryo-maternal interface and to use this model as an *in vitro* diagnostic tool for infertility. Significant progress has been made over the years in generating such models. The first experiments of endometrial modeling involved animal models, which are undoubtedly valuable, but at the same time, their dissimilarities with human tissue represent a significant obstacle to further research. This fact led researchers to develop basic monolayer coculture systems using uterine cells obtained from biopsies and, later on, complex and multilayer coculture models. With successful tissue engineering methods and various cultivation systems, it is possible to form endometrial two-dimensional (2D) models to three-dimensional (3D) organoids and novel assembloids that can recapitulate many aspects of endometrial tissue architecture and cell composition. These organoids have already helped to provide new insight into the embryo-endometrium interplay. The main aim of this paper is a comprehensive review of past and current approaches to endometrial model generation, their feasibility, and potential clinical application for infertility treatment.

## 1 Introduction

The black box is generally defined as a highly intricate and complex system whose inner operation is unknown, hidden, or difficult to understand. This definition perfectly fits the events of early pregnancy. Cimadomo et al. (2023) published a systematic review and meta-analysis in which they thoroughly discussed the problem of the “black box” of early pregnancy loss. They summarized the knowledge gaps about the relatively low success of euploid embryo implantation, from embryo reproductive competence to the intricate interplay between the necessarily thick, immunologically fit, decidualized, and properly receptive endometrium within the window of implantation. Ashary et al. (2018) authored a poetical paper, Embryo Implantation: War in Times of Love. Here, they outlined the whole orchestrated process, which includes the regulation of endometrial receptivity, modification of the luminal epithelium by the embryo, and secretion of embryonic extracellular vesicles, and microRNA, which target and mediate maternal proneness to embryo adhesion, mediating the cross-talk between the embryo and mother.

One of the significant causes of infertility is the failure of the human embryo to implant. This notion has been proved by numerous pregnancy losses happening in the critical early stage of development and by the relatively low rate of successful pregnancies following *in vitro* fertilization (IVF), representing 31% per fresh embryo transfer on average, according to the 2022 estimate by the Human Fertilization and Embryology Authority (HFEA) (Larsen et al., 2013). According to recent hypotheses, implantation failure has a multifactorial background where important roles play immunological dysregulation, disrupted integrity of sperm DNA, and environmental factors.

It was found that the critical detector of embryo quality is decidualized endometrium. Through various molecular pathways, the endometrium seals the fate of a disrupted embryo toward rejection (Albertini, 2023). With the progress in DNA-based methods, the epigenetic regulation of implantation failure is better understood. However, the understanding of the mosaic of endometrium-embryo interactions is still incomplete, so investigating these processes *in vitro* is warranted.

**FIGURE 1:**
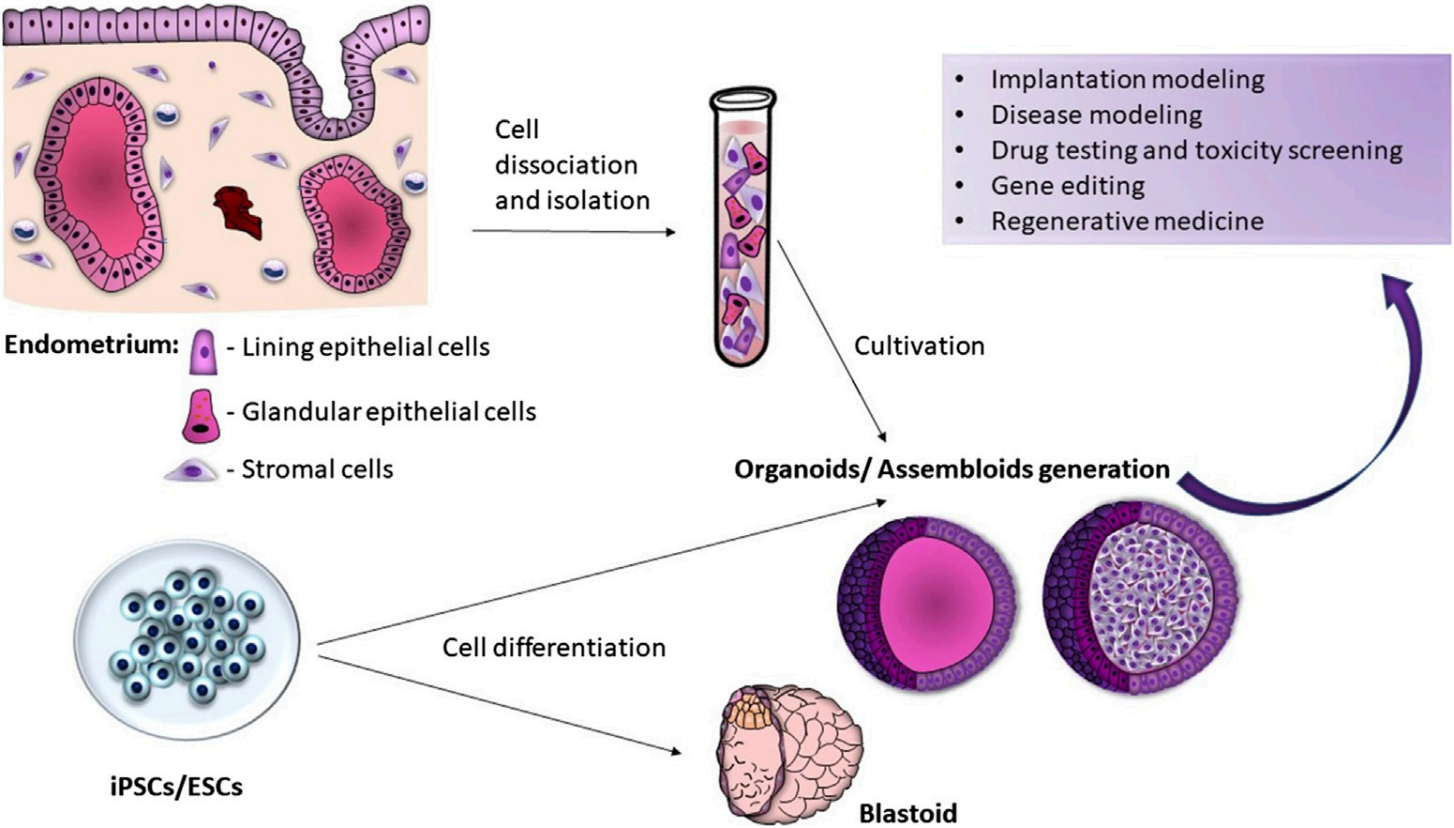
Endometrial organoid/assembloid and blastoid generation.

In the past decade, endometrial model generation has progressed rapidly (Rawlings et al., 2024). Pioneering endometrial biology and pregnancy studies were conducted on *in vivo* animal models, such as murine, ovine, bovine, and non-human primate models. According to extensive results from animal models, researchers were able to utilize many methods used in assisted reproductive technologies (Morrison et al., 2018; Soncin et al., 2018; Boroviak et al., 2018) and even described genes involved in the implantation process, such as leukemia inhibitory factor (*LIF*), *gp130*, interleukin-11 receptor (*IL11R*), and *COX-2* (Stewart et al., 1992; Ernst et al., 2001; Lim et al., 1997; Bilinski et al., 1998). On the other hand, the reproductive tract differences between animal models and humans are significant, as are processes occurring during pregnancy, such as placentation (Lee and DeMayo, 2004; Carter, 2020). Nevertheless, the use of animal models to study implantation *in vivo* is still required due to obvious ethical, legal, and technical issues limiting the availability of human tissue. The breakthrough of *in vitro* models has opened more accessible and convenient alternatives for implantation modeling. Older *in vitro* models were based on mono-layered and multi-layered co-culture systems using primary epithelial or stromal uterine cells, which could not imitate the complex endometrial structure and, therefore, were insufficient (Bentin-Ley et al., 1994; Hopfer et al., 1994; Evron et al., 2011). Many of them lacked extracellular matrix (ECM), a critical component in epithelial differentiation and cellular polarity determination. The mentioned obstacles related to older co-culture systems can be solved through a novel, promising tool–the organoid culture technique (Shibata et al., 2024) (Figure 1).

The present paper provides a detailed overview of endometrial organoids and assembloids generation, their impact on implantation modeling, and possible future clinical applications. The modeling of the fetal part (trophoblast organoids and blastoids) necessary for the study of implantation is also mentioned.

## 2 Endometrial organoids–generation techniques

Organoids have undeniably revolutionized the field of 3D tissue modeling *in vitro*. They are usually derived from pluripotent, fetal, or adult stem cells, differentiated cells, and cancer cells. In a defined 3D environment, these cells can form self-organized mini-clusters and differentiate into viable tissues, or “mini-organs.” They can outline very closely the cellular heterogeneity, structure, and function of the desired tissue and thus contribute to human development and disease modeling. Moreover, organoids are being used in regenerative and personalized medicine, and they are suitable candidates for replacing animal models in toxicity screening and drug testing (Lancaster and Knoblich, 2014; Corrò et al., 2020; Zhao et al., 2022).

As of today, research teams all around the globe have generated numerous organoid types derived from all three germ layers (ectoderm, mesoderm, endoderm), including gastrointestinal organoids (Spence et al., 2011; McCracken et al., 2014; Múnera and Wells, 2017), liver and pancreatic organoids (Hindley et al., 2016; Budi et al., 2024; Wang et al., 2020), kidney organoids (Homan et al., 2019; Vanslambrouck et al., 2023), lung organoids (Kim et al., 2019; Miller et al., 2019), brain organoids (Lancaster et al., 2013; Gabriel et al., 2021; Sun et al., 2022), and many others, including a successful generation of endometrial and tubal organoids (Boretto et al., 2017; Turco et al., 2017; Kessler et al., 2015) as well as blastoids, which represent blastocyst-like structures derived from pluripotent stem cells (Li et al., 2019; Yu et al., 2023).

The endometrium is the uterine mucosa composed of various components such as glandular and lining epithelium (often dubbed as luminal epithelium in most papers) and connective tissue lamina propria featuring stromal, endothelial, and immune cells embedded in the ECM. All these components serve to provide nourishing and an immune-privileged environment to support embryo implantation (Vilella et al., 2021). Therefore, it is evident that a valuable human endometrial model has to contain both stromal elements and epithelial cells. Unfortunately, their long-term co-cultivation has been challenging due to incompatible matrix composition (Song and Fazleabas, 2021; Fitzgerald et al., 2021). This goal was successfully achieved by the Turco et al. (2017) and Boretto et al. (2017). Turco et al. (2017) adapted conditions previously used to generate adult stem cell-derived endometrial organoids and differentiated genetically stable 3D glandular organoid cultures from non-pregnant endometrium and decidua within 7–10 days. Investigators used tissue isolates enriched for epithelial cells as a primary source. Authors stated that these organoids can be expanded every 7–10 days for ˃6 months. Moreover, they formed cystic structures lined by simple columnar epithelium that subsequently started to secrete PAS-positive secretion resembling the appearance of endometrial glands *in vivo*. As a next step, the authors analyzed the global gene expression profile to examine similarities between organoids and donor tissue. The obtained results confirmed the glandular epithelial nature of organoids. Another proof of successful organoid generation was that both responded to sex hormones (estrogen (ES) and progesterone (PG)), demonstrated by immunohistochemistry and microarray analysis. Furthermore, researchers tried to mimic pregnancy by adding placental hormones (human chorionic gonadotropin (hCG) and human placental lactogen (hPL)) to see how the organoids would react. Results showed that the differentiation of human endometrial organoids was further stimulated under the mentioned hormonal treatment. Taken together, Turco et al. (2017) reported an invaluable method to generate a long-term culture of endometrial organoids closely resembling primary endometrial tissue characteristics.

The same year, Boretto et al. (2017) also established novel organoid models from mouse and human endometrium displaying endometrial epithelium physiology and enabling their long-term expansion. The mouse model was made from dissociated mouse endometrium. The glandular-type fragments were further embedded in Matrigel, serving as an ECM scaffold, and cultivated in a medium containing a cocktail of growth signaling factors. Under WNT-activating cultivation conditions, the endometrial fragments started self-organizing and quickly displayed organoid-like structures. Notably, most organoids were formed from endometrium isolated at the estrous phase compared to the metestrus phase. According to the histological analysis of organoid tissue, the lumen was lined by an epithelial layer with microvilli, and glandular fragments with mucous secretion were also detected. In line with the results, the authors stated that WNT3A significantly enhanced the generation and differentiation efficacy of the endometrial epithelial organoid. Mouse endometrial organoids were also responsive to hormonal regulation, namely ES, which increased the proliferation activity of organoid cells, while PG treatment increased mucin production, particularly in immature organoids. All the results mentioned above proved the physiological relevance of these organoid models. Following the success of mouse endometrial organoid generation, the authors decided to develop endometrial organoids from human endometrial biopsies. The basal medium was similar to the previous one used for generating mouse organoids (Sato et al., 2011). Immunohistochemistry revealed the glandular morphology of organoids, the PanCK^+^ layer of epithelial cells covering the lumen, and *FOXA2* marker expression, indicating the presence of glandular epithelium. The apicobasal polarity of epithelial cells was confirmed by observation of apical microvilli and cilia. Lastly, the human organoids were treated with hormones, where ES treatment resulted in more proliferating cells (Ki67^+^ cells), contrary to the effect of progesterone. Glandular epithelium displayed a pseudostratified organization, which is typical of the proliferative phase of the endometrial cycle. On the other hand, the effect of PG led to morphological changes expected in the secretory phase, such as folded and coiled glands lined by columnar epithelium with intracellular subnuclear vacuolation and increased mucin production. Regarding the results, researchers generated mouse and human organoids, which could mimic cycle-specific endometrial changes in response to hormones, thus representing another promising endometrial model.

The research group of Haider et al. (2019) generated endometrial organoids to explore the effect of ES on the cell fate of human endometrial epithelium. As a primary cell population, human decidual cells and several stromal cells were used to form organoids. Light and transmission electron microscopy (TEM) confirmed the presence of cystic structures lined by simple columnar epithelium with microvilli on their apical surface and secretory vesicles together with glycogen granules in the cytoplasm resembling characteristics of human decidua. The ES treatment also triggered cilia formation. Based on these results, the authors concluded that ES is a vital factor in triggering the process of ciliogenesis.

Fitzgerald et al. (2019) generated self-renewing endometrial organoids from human endometrial biopsies by a similar protocol within 3–4 days. Moreover, the authors identified multiple cell types within organoids according to gene expression involving ciliated epithelial, glandular, stem, and unciliated epithelial cells for the first time. Interestingly, the number of epithelial and stem cells decreased under ES treatment.

All other following endometrial organoid generation studies have been based on the protocols mentioned above with minor modifications involving the addition of supplements, such as insulin-like growth factor (IGF) (Boretto et al., 2019), N-Acetyl-L-Cysteine (Luddi et al., 2020), N2, B27, and Primocin (Abbas et al., 2020; Tang et al., 2023), Rock inhibitor Y-27632 (Gnecco et al., 2023; Shibata et al., 2024), Jagged-1 (Maru et al., 2019), and hydrocortisone and heparin (Murphy et al., 2019; Wiwatpanit et al., 2020) (Table 1).

**TABLE 1:** Overview of selected cultivation methods used for uterine organoids.

Cell source	Medium components	Use of matrigel	Species	Features of organoids	Ref.
Murine and human endometrial cells	WNT3A, R-spondin 1, EGF, FGF10, Noggin, A83-01, ITS; N-acetyl-L-cysteine and SB202190	yes	Murine, human	Expression of glandular epithelial markers; response to steroid hormones; mucin production; coiled glands lined by simple columnar epithelium	Boretto et al. (2017)
Murine and human non-pregnant endometrium and decidua	EGF, HGF, FGF10, R-spondin 1, Noggin, A83-01, nicotinamide	yes	Murine, human	Expression of glandular epithelial markers; PAS- positivity of secretion; response to steroid hormones	Turco et al. (2017)
Human decidual and endometrial stromal cells	Noggin, A83-01, R-spondin, EGF, and CHIR99021	yes	human	Presence of cystic structures lined by simple columnar epithelium with microvilli; cytoplasmatic glycogen granules; ciliogenesis during proliferative phase	Haider et al. (2019)
Human endometrial epithelial and stromal cells	Similar to Turco et al. (2017) with the addition of Y-27632	yes	human	Identification of several endometrial cell types; response to steroid hormones; expression of epithelial markers	Fitzgerald et al. (2019)
Human endometrial epithelial and stromal cells	Growth medium (MammoCult™), hydrocortisone and heparin	no - instead agarose 3D Petri Dishes	human	Clusters of cells exhibit endometrium characteristics; epithelial cell polarization PAS- positivity of secretion; response to steroid hormones	Wiwatpanit et al. (2020)
Human endometrial epithelial cells	Similar to Turco et al. (2017) with the addition of N-Acetyl-L-cysteine	yes	human	Apical polarization of pseudostratified epithelium - presence of microvilli; accumulation of glycogen and mucus; response to steroid hormones	Luddi et al. (2020)
Human decidual stromal cells and endometrial epithelial cells	Similar to Turco et al. (2017) with the addition of N2, B27, and Primocin	no - instead porous collagen scaffold	human	Epithelial cells with microvilli and cilia; the presence of glycocalyx and lipid droplets; response to steroid hormones	Abbas et al. (2020)
Human endometrial epithelial and stromal cells	Y-27632	no - instead synthetic (PEG) ECMs	human	Recapitulation of hormone-induced changes similar to a 28-day menstrual cycle	Gnecco et al. (2023)

In 2020, a research team (Abbas et al., 2020) developed an advanced approach to endometrial organoid generation. The authors built up a multicellular model based on a porous collagen scaffold with controlled lyophilization containing epithelial cells from biopsies of the endometrium at the secretory phase and stromal cells of decidual tissue. Cell cultivation was similar to that of previous studies. This novel study enabled the replacement of artificial ECM, such as Matrigel or GelTrex, with ECM, including collagen bundles produced by organoid cells, which is much more favorable. Unfortunately, the authors did not achieve glandular organization within organoids.

Later on, other investigators developed alternatives to artificial ECM as well. Jamaluddin et al. (2022) fabricated hydrogels from decellularized human and bovine endometrium, which displayed better properties regarding the support and growth of endometrial organoids than those embedded in Matrigel.

Regardless of the success of hydrogels obtained from decellularized human endometrium, the limited tissue availability, ambiguous reproducibility, rapid ECM breakdown, and intra-patient differences still represent a considerable challenge. Currently, the interest is focused on developing fully defined synthetic matrix scaffolds in which composition and biomechanical properties could be precisely controlled (De Vriendt et al., 2023). Recently, Gnecco et al. (2023) engineered synthetic matrices made from polyethylene glycol (PEG)-based hydrogel cross-linked with matrix metalloproteinase-labile peptides. These new tissue-inspired synthetic ECMs enabled the co-cultivation of epithelial and stromal cells by targeting their cell-specific integrins and, therefore, mimicking the biophysical properties of the endometrium. However, the authors mentioned several limitations, such as the performance of assays at the endpoint of measurements and the need for future mechanistic studies.

It is important to note that adding immune cells to the endometrial organoid model is also a necessary prerequisite for creating a proper endometrial environment. For instance, uterine natural killer (uNK) cells, upon their activation, release essential cytokines to protect the host from pathogens, support decidualization and spiral artery remodeling, remove senescent decidual cells, and contribute to immunity tolerance and fetal development (Xie et al., 2022).

## 3 Endometrial assembloids–generation techniques

Compared to organoids, assembloids are generally defined as self-organizing 3D culture systems, which are more complex by combining various organoids within one functional framework. While organoids generally are self-organized 3D models reflecting a specific functional aspect of a given organ by modeling functional-morphological intricacies of several cellular components and tissue constituents, assembloids integrate multiple such systems to more closely mimic the real *in vivo* interactions (Kanton and Paşca, 2022). In recent years, various research teams have generated endometrial assembloids (Rawlings et al., 2021a).

It is important to note that the terminology is sometimes confusing. While the previous authors considered 3D gland-like structures organoids, and by adding stromal elements, they termed these composite objects assembloids, Shibata et al. (2024) generated an epithelial structure called apical-out endometrial organoid (AO-EMO), which was later combined with stromal cells (SCs), as well as self-formed endothelial elements. Only after these organoids were combined with human embryonic stem cell-derived blastoids the authors used the term assembloid. Tian et al. (2023) pointed out that a simple combination and culture of epithelial and stromal cells do not fully represent all the complexities of endometrial functional morphology. They referred to these simple aggregates as assembloids (EnAOs) and provided a framework for creating more complex assembloids. The main limitation of the EnAOs is the absence of critical structural features of the endometrium, such as the lining epithelia-resembling structure (luminal epithelium) and the lack of epithelial gland-like structures. To address this, the authors utilized an air-liquid interface (ALI) culture, submerging the basal cell surface in liquid. At the same time, the apical portion is exposed to air, mimicking the organization of epithelial cells *in vivo*. Comparative analysis showed that this novel approach best replicates endometrial functional morphology, including typical structure, menstrual cycle changes, and gene expression patterns observed during the implantation window.

## 4 Trophoblast organoids and blastoids–generation techniques

Investigating both sides of the equation is vital to studying the fetal-maternal interactions during implantation. Focusing on the fetal/embryonic side, scientists had to develop models of the early stages of development from the embryo perspective (Liu and Polo, 2024). One approach is the generation of trophoblast organoids. Turco et al. (2018) derived trophoblast organoids from human placental tissue. Next, trophoblast organoids were used to generate extravillous trophoblast. The authors could develop a complex 3D structure anatomically and functionally resembling the placenta *in vivo*.

There are also models called blastoids, which can be generated using embryonic stem cells harvested from the inner cell mass, as reported by Kagawa et al. (2022). However, this approach only reiterates the same ethical problem of using any tissue or cell whatsoever that formed after fertilization. Therefore, induced pluripotent stem cells (iPSCs) are perfect for this purpose. Fan et al. (2021) implemented a 3D two-step differentiation protocol that involved the conversion of iPSCs into extended pluripotency stem cells (EPSs). Subsequently, EPSs were used to generate EPS-blastoids, which formed on Day 5. Finally, the authors performed immunofluorescence and scRNA seq analysis, which showed that EPS-blastoids are similar to human blastocysts, making them a suitable modeling tool for implantation research. Using a similar approach, Liu et al. (2021) reprogrammed fibroblasts into iPSCs and then into iBlastoids. By the same token, Yu et al. (2021) harvested human foreskin fibroblasts, reprogrammed them into iPSCs, and generated human blastoids. All authors concluded that this approach is ethical, scalable, and suitable for basic and translational research.

## 5 Endometrial organoids and assembloids–implantation modeling

Implantation modeling is crucial in fully understanding fetal-maternal interaction during early pregnancy. Despite the advancements in IVF techniques, the lack of a detailed understanding of implantation is one of the principal limitations of IVF success (Aghajanova, 2020). From the evolutionary perspective, spontaneous decidualization of the endometrium occurs in a relatively minor group of species, including humans. It reflects the need for complete maternal control over the implantation and hemochorial placentation, which pose significant immunological challenges (Gellersen and Brosens, 2014). Although Hertig et al. (1956) studied human implantation directly in hysterectomy samples using histological methods, such studies are rare. There are also ethical concerns. Even though some countries allow IVF-generated excess pre-implantation embryos for research purposes, once the blastocyst is implanted, its direct analysis is challenging (Fu et al., 2021). The first and most logical step to overcome this hurdle is to use animal models. As previously mentioned, the differences between humans and other species limit their application (Rawlings et al., 2021b). Therefore, a novel approach uses the *in vitro* culture of human cells in different layers of complexity, from monolayer culture to layered co-culture to 3D organoid/assembloid models.

### 5.1 Overview of modeling approaches to implantation

Blastocyst implantation can be modeled in a plethora of different ways. As comprehensively reviewed by Ojosnegros et al. (2021), there are *in vitro* models using primary endometrial epithelial culture, immortalized epithelial cell lines, Ishikawa cells, low receptivity HEC-1-A cells, high receptivity RL95-2 cells, ECC-1 cells derived from endometrial carcinoma, *in vitro* systems using hTERT-EECs, or primary and immortalized ESC lines. Next are decidualization models, which are achieved by hormonal stimulation of ESCs. Modeling is also performed by preparing embryo surrogates like trophoblastic cell lines and spheroids. A more complex system includes Transwell assays. All these systems can provide valuable information, but their general setting is the attachment or invasion of embryo surrogates to a 2D monolayer of different characteristics.

As reviewed by Kim and Kim (2017), many aspects of implantation are partially understood thanks to these models, including apposition, adhesion, and early blastocyst invasion. Implantation modeling *per se* has been in progress for several decades. Based on the preliminary knowledge of the implantation process from the 1950s, Kliman et al. (1990) developed an *in vitro* suspension co-culture system to study adhesive interactions between trophoblast cells and endometrium. One of the main findings of this study was that trophoblast attachment crucially depends on intact endometrium. Galán et al. (2000) established a 2D model that demonstrated that at the implantation site, coordinated apoptosis of endometrial epithelial cells occurs. The authors were the first to show that blastocyst induces apoptosis of the epithelial cells in a paracrine manner based on the Fas/Fas-L interaction. Considering the drawbacks of 2D modeling, 3D approaches have emerged, providing a more precise recapitulation of *in vivo* conditions. One of the initial 3D approaches to implantation modeling has been the implementation of less complex spheroids (compared to organoids and assembloids). These have been prepared from the human choriocarcinoma Jar cell line with 3D endometrial cultures (Wang et al., 2012). The same research team established another similar model, which enabled the study of hormone-dependent interactions between epithelial and stromal cells and the attachment and invasion of stromal cells (Wang et al., 2013). Trophoblast invasion has also been modeled by establishing an interaction between trophoblast cells and gland-like endometrial spheroids (Buck et al., 2015). Stern-Tal et al. (2020) used Jar spheroids as embryo models attached to 3D macroporous alginate scaffolds seeded with endometrial epithelial cells. This study showed that E-cadherin expression is directly proportional to endometrial receptivity.

### 5.2 3D models using organoids and assembloids

Rawlings et al. (2021a) used their generated endometrial assembloid to study embryo implantation in decidual senescence. Senescence is defined as a state of metabolic activity without active cell division. The authors constructed a simple model of embedding a human embryo in their endometrial assembloid. The principal experimental input was the application of dasatinib, a tyrosine kinase inhibitor that almost totally eliminates senescent decidual cells. Senescence is essential in implantation because when it is too exaggerated, it can lead to recurrent miscarriage, while lacking senescence may be a causative factor in implantation failure. The main discovery of this study was that dasatinib-induced elimination of senescent decidual cells led to the entrapment of embryos in a stagnant decidual matrix, which hindered successful implantation. Another important finding was that endometrial assembloids could be used to model various stages of implantation and may serve as models for testing drugs that help prevent reproductive failure. However, a disadvantage of these assembloids is that they do not precisely resemble the endometrium because they lack the lining epithelium and immune cells within the stromal compartment.

Organoids are also useful for modeling the interaction of maternal uterine Natural Killer (uNK) cells, which comprise around 70% of all white blood cells in the decidua (Lapides et al., 2023). These cells are located near infiltrating trophoblast and maternal spiral arteries, suggesting they play a role in regulating the extent of trophoblast invasion for proper placentation. Disruption of this interaction may be a causative factor in recurrent implantation failure, habitual abortion, and idiopathic sterility (Lapides et al., 2022a). In a recent study, Li et al. (2024) used a trophoblast organoid to model the role of uNK cells in placentation by exposing trophoblast organoids to a panel of cytokines produced by uNK cells. The subsequent transcriptomic analysis showed that uNK cytokines stimulate the differentiation of trophoblast cells into extravillous trophoblast (EVT), which is crucial for adequately remodeling maternal spiral arteries and developing normal fetal-maternal circulation. The research team also found that uNK cells suppressed innate or adaptive immune responses, which is consistent with their previous investigations on this topic (Vento-Tormo et al., 2018). The organoid model also showed that uNK cells may promote fetal growth by increasing blood flow and nutrient access. This study also outlined a possible link between uNK-mediated placentation and disorders such as preeclampsia, fetal growth restriction, and placenta accreta. These are the reasons why histological assessment of the endometrium (currently using mini-invasive techniques) has become a part of complex diagnostic and therapeutic approach in selected women with infertility (Lapides et al., 2022b).

It has been long known that endometrial glands are more than just a source of nutritious substances. They upregulate various genes necessary for implantation. For instance, the secreted phosphoprotein 1 (SPP1) (osteopontin)-encoding *SPP1* gene which facilitates adhesion of the blastocyst to the epithelial lining, LIF-encoding *LIF* gene which is involved in adhesion, embryo development, and trophoblast differentiation, or progestogen-associated endometrial protein (PAEP) (glycodelin)-encoding *PAEP* gene which contributes to cross-talk between the implanting blastocyst and luminal epithelium. Endometrial glands also produce several growth factors–epidermal growth factor (EGF), vascular endothelial growth factor (VEGF), and transforming growth factor beta (TGFB). Their respective functions are stimulation of cytotrophoblast proliferation and syncytiotrophoblast secretion of human chorionic gonadotropin (hCG) and human placental lactogen (hPL) by EGF, trophoblast adhesion to the luminal epithelium by VEGF and ECM remodeling by TGFB (Rawlings et al., 2021b). The generation of glandular organoids has tremendous potential in further elucidating these critical processes involving endometrial glands. Turco et al. (2017) demonstrated that their organoids responded to sex hormones estradiol (E2) and progesterone (P4), and their further stimulation by hCG, hPL, and prolactin resulted in PAEP (glycodelin) and SSP1 (osteopontin) secretion by the organoid. The authors concluded their organoid will be a precious tool not only for further research of implantation but also for therapy testing and the investigation of histiotrophic nutrition. Luddi et al. (2020) published an experimental study that involved the endometrial organoid generation and a subsequent study of critical features of the implantation window. Their organoid model corroborated that glycodelin A is an important marker of endometrial receptivity. One of the paper’s key findings was that hormonal treatment affects not only glycodelin A-encoding *PAEP* gene expression but also glycodelin A glycosylation pattern. Glycosylation of glycodelin A significantly affects its biological activity, as different patterns of glycosylation have been linked to different aspects of implantation and placentation like blastocyst attachment, trophoblast differentiation, hormonal regulation, trophoblast invasion, placental angiogenesis, and promotion of immune tolerance (Lee et al., 2016). Moreover, Luddi et al. (2020) used transmission and scanning electron microscopy (TEM and SEM, respectively) to demonstrate that their organoid closely resembled *in vivo* glandular morphology in different menstrual cycle phases. On top of that, the SEM investigation revealed that upon induction of the organoid into the secretory phase, its luminal surface formed pinopodes - apical cell membrane protrusions of the uterine epithelial cells that are suggested as reliable crucial indicators of the implantation window.

In one of the most recent papers, authored by Shibata et al. (2024), their assembloid consisting of AO-EMO co-cultured with a blastoid (already described above) successfully addressed various downsides of conventional organoids by ensuring apical accessibility, fixing stromal cell shortage, and vascularization issues. This model closely recapitulated different implantation stages, including apposition, adhesion, and invasion. The principal implantation insights gained from the assembloid model used in this novel study can be summarized as follows: blastoid displayed polar adhesion, indicating that endometrial epithelium is a crucial regulator of normal embryo adhesion. Next, the adhesion rate was diminished in the absence of stromal cells, confirming the important role of a high density of endometrial stromal cells for implantation to occur properly. This finding couldn´t have been achieved in traditional flat models due to gel contraction by abundant stromal cells in such settings. Another finding was that endometrial epithelium promotes syncytiotrophoblast differentiation, which is crucial in breaching the epithelial barrier necessary for invasion. The study also revealed that syncytial cells fuse with stromal cells, evidenced by the disappearance of stromal cell marker vimentin upon fusion, which probably influences the extent of embryo invasion. Finally, yet importantly, there are also trophoblast and vascular organoids that can be used to model ectopic pregnancies (Zhao et al., 2023).

## 6 Endometrial organoids and assembloids–disease modeling

Endometrial dysfunction is responsible not only for implantation failure but also for the onset of numerous other disorders, such as endometriosis and endometrial cancer. Since endometrial cancer is prevalent and its occurrence has increased tendency even in younger patients (Repiska et al., 2010), other experiments by Turco et al. (2017) were focused on the generation of endometrial organoids from samples of endometrial adenocarcinoma. These organoids were similar to the primary tumor in several aspects involving pleomorphic cells, disorganized epithelium, and SOX17 positivity so that they can serve as a good model for drug therapy and investigation of mutational changes.

A more complex study was published by Boretto et al. (2019), who established patient-derived endometrial cancer organoids from different samples of low to high-grade cancer, as well as organoid models of endometriosis and hyperplasia (simple benign, complex atypical, polyp). All generated organoids replicated the disease’s phenotypes and genetics. For example, precancerous organoids displayed PanCK^+^ epithelium lining the lumen. Interestingly, endometrial cancer organoids (EC-O) showed limited proliferative capacity and normal genome; therefore, the authors adjusted culture conditions and removed p38i from the basal medium. Afterward, EC-O showed a grade-associated degree of abnormalities regarding the nuclear changes, expression of tumor-associated markers and ion channel markers, disease-specific gene expressions, and many new substitutions after long-term expansion. Moreover, EC-O also exhibited disease phenotype *in vivo* after their transplantation under the kidney capsule of NOD-SCID mice.

Similarly, Maru et al. (2019) developed patient-derived organoids from endometrial and ovarian tumor samples. All generated organoids retained histological and genetic features of the original tumor. Namely, the EC-O displayed cribriform or dense structure for endometrial endometrioid carcinoma grade 2 or loosely cohesive cancer cell clusters with nuclear atypia for endometrial carcinoma grade 3. In their most recent study, Maru et al. (2024) established multiple patient-derived organoids from uterine carcinosarcoma tissue samples and used them for drug screening analyses. Results identified four reagents that were effective for all EC-O. Bi et al. (2021) constructed patient-derived organoids from patients with recurrent endometrial cancer, aiming to predict sensitivity to chemotherapy with neoadjuvant trastuzumab. As it was found out using the EC-O model, the patient was resistant to this agent. Additionally, the researchers were able to identify alternative treatment possibilities. The abovementioned study and a few others (Girda et al., 2017; Chen et al., 2021; Chen X. et al., 2023; Sengal et al., 2023) underline the therapeutic potential of EC-O in novel drug screening and drug sensitivity and toxicity testing.

The pathologies behind polycystic ovary syndrome (PCOS) were studied on an endometrial organoid developed by Wiwatpanit et al. (2020). Scaffold-free organoids were derived from primary endometrial epithelial cells and stromal cells and were treated with estradiol and testosterone for 14 days to mimic PCOS. It was found that the mentioned hormonal treatment increased cell proliferation (spindle microtubules) and caused gene dysregulation of the endometrial organoid model. Furthermore, gene ontology analysis revealed the involvement of diverse neoplasms, such as endometrial and urogenital, which can finally lead to tumor development. Taken together, the authors successfully developed an endometrial organoids model of PCOS and showed that excessive androgen treatment directly impacts endometrial cells.

Another exciting application of endometrial organoids derived from endometrial HEC1A cancer cells came from a research group of Łaniewski et al. (2017) interested in studying uterine epithelial-microbiota interactions. Authors infected 3D organoids with pathogenic bacteria involving *Lactobacillus crispatus, Gardnerella vaginalis*, and *Neisseria gonorrhoeae.* All microbiota colonized organoid models; however, only *Neisseria gonorrhoeae* triggered a significant proinflammatory reaction accompanied by ultrastructural tissue changes. The presented 3D model could recapitulate the host-microbe interaction, which had not been previously observed in endometrial organoid models. Using a similar organoid infection model, Dolat and Valdivia (2021) studied the cell biology of *Chlamydia trachomatis* infection. The authors observed that *C. trachomatis* induced the reorganization of the cytoskeleton and Golgi apparatus. Furthermore, the infected model enabled the recapitulation of processes involved in cellular immunity, such as the expression of neutrophil chemoattractants.

## 7 A brief overview of other female reproductive system organoids

Over the past few decades, apart from endometrial organoids, research groups have also generated “mini-organs” resembling other tissues of the female reproductive system. For instance, ovarian organoids were generated primarily as an ovarian carcinoma model or as a 3D platform for pre-clinical drug testing (Kopper et al., 2019; Maenhoudt et al., 2020; Chang et al., 2022; Fashemi et al., 2023). In the mentioned studies, the ovarian carcinoma organoids reflected disease pathophysiology and morphological characteristics. Regarding drug testing, thanks to ovarian cancer organoids generated by the group of Gray et al. (2023), a patient suffering from low-grade serous ovarian cancer received personalized targeted therapy, including ibrutinib. Selected off-label monotherapy resulted in remarkable turnover over the following 65 weeks. Another research group identified therapeutic options for stage IV ovarian carcinoma involving the combination of fulvestrant with everolimus, emphasizing the importance of such organoid models in clinical practice (Al-Aloosi et al., 2023).

Uterine tube (UT) organoids have also been generated to model various, primarily oncological, conditions. Chang et al. (2020) derived UT epithelial cells (UTECs) from human UTs. UTECs were put into Matrigel, and an organoid was generated after co-culture with stromal and endothelial cells. The authors concluded that such a model can be useful for regeneration and malignant transformation modeling. Yucer et al. (2021) developed an *in vitro* model of *BRCA1* mutation using iPSCs-derived organoids. This model proved to be a suitable and faithful model of early carcinogenesis of high-grade serous ovarian cancer. Another model of this pathological condition, but with a focus on different mutations, namely *TP53* and *RAD51D*, was recently reported by Dai Y. et al. (2024). The research group generated an organoid from primary human UTECs and found it invaluable in studying these mutations’ effect on the disease pathogenesis. They also highlighted their future potential in drug screening. One of the reasons to model the UT *in vivo* is to broaden the knowledge of immune cells, which, within the UT epithelial lining, suppress immune reactions against sperm and embryo, making the UT an immune-privileged organ (Visnyaiová et al., 2024).

## 8 The future of endometrial 3D modeling

Without hesitation, the development of endometrial organoids or assembloids is still in its infancy. At the same time, it bears enormous potential in the field of implantation modeling and exploration of various endometrial disease pathologies.

During the last 7 years, the endometrial organoid generation, implantation, and disease modeling experiments led by many research groups have experienced exponential growth. However, we are still far from the ultimate goal, which represents the development of a 3D construct able to recapitulate the cellular complexity of the endometrium *in vivo* involving both luminal and glandular epithelium, endometrial stromal cells, and immune cells (Guo et al., 2023).

Several challenges need to be solved to reach the goal mentioned above. One of them is the cultivation method. The cultivation protocols used for endometrial organoid generation vary greatly, indicating the inconsistency in generation techniques. Usually, primary cell populations for organoid generation are obtained from endometrial biopsies. However, Cindrova-Davies et al. (2021) investigated an easier way to obtain endometrial cells - menstrual blood. Interestingly, glandular fragments within menstrual blood formed menstrual organoids displaying similar features as endometrial organoids regarding proliferation rate, derivation efficiency, response to sex steroid hormones, and transcriptomic signature. Therefore, menstrual organoids represent a promising personalized, non-invasive cell harvesting technique. Another source of endometrial cells could be those differentiated from iPSCs. Endometrial stromal cells were generated for the first time from iPSCs by the research group of Miyazaki et al. (2018). The differentiation process started from intermediate mesoderm to coelomic epithelium formation and, subsequently, the Müllerian duct. The endometrial stromal fibroblasts within embryoid bodies were observed on day 14. Undeniably, iPSC-differentiated endometrial cells bring new hope to the field of patient-specific endometrial regeneration and personalized disease modeling.

Another challenge is replacing the widely used Matrigel as an ECM for organoid cultivation due to its long-term instability and the insufficient similarity of the human endometrial niche (Gu et al., 2020). Luckily, several recent studies came up with other alternatives, such as porous collagen scaffolds (Abbas et al., 2020), artificial hydrogels from decellularized human endometrium (Jamaluddin et al., 2022), or engineered synthetic PEG hydrogels (Gnecco et al., 2023). Embedded within PEG hydrogel, the endometrial stromal cells could differentiate into decidual cells under hormonal stimulation (Cook et al., 2017). In the most recent article, Salisbury et al. (2024) reported the development of semisynthetic gelatine methacryloyl hydrogel (GelMA) that combines bioactive properties together with the advantages of synthetic material, such as reproducibility and tunability. The constructed photo-cross-linked gelatine-based hydrogel supported the development of endometrial organoids; specifically, it maintained the viability of stromal cells and upheld epithelial gland formation. Besides that, the GelMA has potential in bioprinting thanks to the similar wavelength of the UV light, which was needed for its fabrication. Zambuto et al. (2024) broadened the application possibilities of such GelMA by engineering an endometrial microvascular network embedded in the gelatin-based hydrogel. The endometrial microvascular network displayed patterns typical for decidualization and influenced the trophoblast cells; however, trophoblast cells did not induce structural changes of engineered microvasculature. The research helped to shed light on uterine-trophoblast interface.

Despite the abovementioned issues regarding the generation of endometrial organoids, the vast majority of developed organoids responded to hormonal treatment (ES, PG), recapitulating changes of luminal and glandular epithelium from the proliferative to mid-secretory phase. Gene analyses of endometrial organoids in the mid-secretory phase also reflected the increased expression of genes specific to this phase, including *LIF, HSD17B2, PAEP, GPX3*, and *FOXO1* (Turco et al., 2017; Vasquez et al., 2018; Fitzgerald et al., 2019).

The generation of assembloids made an important step forward in endometrial organoid research (Tian et al., 2023; Rawlings et al., 2024). According to researchers, adding other key cell types, such as endothelial and immune cells, is necessary to establish more endometrium-like organoids. The most recent study by Shibata et al. (2024) describes the generation of unique AO-EMO with the ability to form its own endothelial network from human umbilical vein endothelial cells. Such assembloids, in the presence of blastoids, could mimic several phenomena occurring between embryo and endometrium.

Endometrial microfluidic culture systems are worth mentioning as another approach to investigating cellular interactions within the endometrium (Dai W. et al., 2024). The microfluidic organ platform, the so-called “Organ-on-Chip,” enables cells and tissue to mimic organ physiology while increasing the complexity of the experimental system. The physiologic conditions are controlled by fluid shear force, concentration gradients, and dynamic mechanical stress (Campo et al., 2020). These systems can also recapitulate parenchymal-vascular interactions, as was confirmed by Gnecco et al. (2019). Authors co-cultured human endometrial stromal cells with primary uterine microvascular endothelial cells within a microfluidic endometrial model. The researchers significantly enhanced decidualization and stromal cell differentiation through hormonal influence and hemodynamic forces, indicating that the vascular endothelium is a substantial physiologic player during decidualization. More recently, Ahn et al. (2021) improved the above-mentioned model by adding a third cell population, namely the endometrial endothelial cells, and created a micro-engineered vascularized endometrium-on-chip consisting of three layers: epithelium, stroma, and blood vessels. According to the authors, this microfluidic endometrial model recapitulated *in vivo* endometrial vasculogenesis and angiogenesis and responded to hormonal treatments mimicking events of the proliferative and secretory phases of the menstrual cycle. Apart from its suitability for implantation modeling, this device was also found to be useful for emergency birth control drug levonorgestrel testing. Despite the advantages of these endometrial microfluidic systems, the investigation of cell-to-cell communications is still hindered by several limitations, such as inadequate calculation of exact shear stress rates within the microvasculature similar to human endometrium, which leads to impaired blood flow. Another limitation is the maintenance of long-term *in vitro* culture, which is also a problem for other 3D models (Gnecco et al., 2019; Deng et al., 2024).

Nowadays, 3D endometrial models involving organoids, assembloids, and microfluidic systems are used in clinical practice mostly for disease modeling, such as endometrial cancer and drug screening, indicating their enormous potential for pharmacological experiments, which had been already proven by several studies (Girda et al., 2017; Boretto et al., 2019; Maru et al., 2019; Bi et al., 2021; Su et al., 2023; Chen J. et al., 2023).

Additionally, the success of gene editing methods, such as CRISPR tools, can be used to study the role of specific gene mutations in endometrial pathologies and the effects of particular gene absence on endometrial functions. The proper application of CRISPR/Cas9 genome engineering in organoids is believed to bring personalized regenerative medicine closer to clinical application (Geurts et al., 2023).

A difficult task keeping the scientific community busy at this moment is to identify an endometrial organoid most suitable for *in vitro* implantation. It is important to mention that the progress regarding the fetal part (blastoid) models of implantation research is certainly significant, but it also faces several challenges. Fortunately, many previous downsides of blastoid modeling, including low efficiency, have been recently addressed, making them more suitable for future modeling of fetal-maternal cross talk. Yu et al. (2023) were able to produce high-fidelity human blastoids from naïve pluripotent stem cells on large scales. This achievement enabled them to facilitate their broader use in a more accessible and scalable manner.

In the future, endometrial organoids should also be implemented to study additional implantation-related processes that are only partially understood. A great potential is the future organoid modeling of blastocyst quality assessment by the endometrium. Various authors previously suggested that endometrium reacts to molecular cues from the blastocyst, assessing its high or low quality, which results in enhanced or decreased receptivity. For example, Teklenburg et al. (2010) co-cultured decidualized endometrial stromal cells (ESCs) with blastocysts and found that ESCs are biosensors of embryo quality, preventing maternal investment into embryos with a low chance of successful development. Establishing an endometrial organoid model to further study this cross-talk is even more important in light of the hypothesis that recurrent miscarriage may not be the result of some obscure condition but can be rooted in the failure of the “quality sensing” mechanism, which enables unfit embryos to implant long enough to produce clinical pregnancy, even though they are destined to perish (Larsen et al., 2013).

## 9 Conclusion

Organoids and assembloids are state-of-the-art advanced 3D models that can closely replicate critical stages of human embryo implantation, providing invaluable knowledge. They can also be effectively used for disease modeling and drug testing. However, before organoids and assembloids can be fully implemented in the clinical setting, several challenges and drawbacks must be addressed in future research. The first issue is that organoids and assembloids are complex, which makes standardization difficult. There are many variables in organoid formation, such as differences in size, shape, and cellular composition, which can affect reproducibility. Secondly, many microenvironmental factors are hard to emulate exactly *in vitro*. For example, the complexity and dynamics of hormonal influences and immune cell and stromal cell interactions make the full physiological relevance of such models ambiguous. Another issue is that most organoids lack a vascular system, which limits their long-term viability. Despite circumventing many ethical concerns within basic research, clinical applications will possibly pose new concerns. Scalability and the cost of these models are also problems, which will be more pronounced when attempting to apply them to large-scale studies and therapeutic applications. Lastly, the bench-to-bedside application will require robust validation and demonstration of clinical relevance, which will be expensive and time-consuming. Overcoming these challenges will require improved standardization protocols and interdisciplinary collaboration. These attempts to form reproducible generation and application guidelines have already been implemented (Ahn et al., 2024).

When future research endeavors address all the aforementioned challenges, they will become even better tools for understanding the critical stages of human development and will open new possibilities for even more effective assisted reproduction techniques with better outcomes for patients.
