# Supplementary material for: Exploring the black box of human reproduction: endometrial organoids and assembloids - generation, implantation modeling, and future clinical perspectives
Source: Front Cell Dev Biol. 2024 Oct 23;12:1482054. doi: 10.3389/fcell.2024.1482054 (PMC11539068; doi:10.3389/fcell.2024.1482054)
Supplement: Supplementary file 1 [file Table1.DOCX]

Supplementary Material

# Supplementary Table

**Table 1. Overview of selected cultivation methods used for uterine organoids**

| **Cell source** | **Medium components** | **Use of Matrigel** | **Species** | **Features of organoids** | **Ref.** |
| --- | --- | --- | --- | --- | --- |
| Murine and human endometrial cells | WNT3A, R-spondin 1, EGF, FGF10, Noggin, A83-01, ITS; N-acetyl-L-cysteine and SB202190 | yes | Murine, human | Expression of glandular epithelial markers; response to steroid hormones; mucin production; coiled glands lined by simple columnar epithelium | Boretto et al. (2017) |
| Murine and human non-pregnant endometrium and decidua | EGF, HGF, FGF10, R-spondin 1, Noggin, A83-01, nicotinamide | yes | Murine, human | Expression of glandular epithelial markers; PAS- positivity of secretion; response to steroid hormones | Turco et al. (2017) |
| Human decidual and endometrial stromal cells | Noggin, A83-01, R-spondin, EGF, and CHIR99021 | yes | human | Presence of cystic structures lined by simple columnar epithelium with microvilli; cytoplasmatic glycogen granules; ciliogenesis during proliferative phase | Haider et al. (2019) |
| Human endometrial epithelial and stromal cells | Similar to Turco et al. (2017) with the addition of Y-27632 | yes | human | Identification of several endometrial cell types; response to steroid hormones; expression of epithelial markers | Fitzgerald et al. (2019) |
| Human endometrial epithelial and stromal cells | Growth medium (MammoCult^TM^), hydrocortisone and heparin | no  - instead agarose 3D Petri Dishes | human | Clusters of cells exhibit endometrium characteristics; epithelial cell polarization PAS- positivity of secretion; response to steroid hormones | Wiwatpanit (2020) |
| Human endometrial epithelial cells | Similar to Turco et al. (2017) with the addition of N-Acetyl-L-cysteine | yes | human | Apical polarization of pseudostratified epithelium - presence of microvilli; accumulation of glycogen and mucus; response to steroid hormones | Luddi et al. (2020) |
| Human decidual stromal cells and endometrial epithelial cells | Similar to Turco et al. (2017) with the addition of N2, B27, and Primocin | no  - instead porous collagen scaffold | human | Epithelial cells with microvilli and cilia; the presence of glycocalyx and lipid droplets; response to steroid hormones | Abbas et al. (2020) |
| Human endometrial epithelial and stromal cells | Y-27632 | no  - instead synthetic (PEG) ECMs | human | Recapitulation of hormone-induced changes similar to a 28-day menstrual cycle | Gnecco et al. (2023) |
