# Supplementary material for: Exploring the black box of human reproduction: endometrial organoids and assembloids - generation, implantation modeling, and future clinical perspectives
Source: Front Cell Dev Biol. 2024 Oct 23;12:1482054. doi: 10.3389/fcell.2024.1482054 (PMC11539068; doi:10.3389/fcell.2024.1482054)
Supplement: Supplementary file 2 [file DataSheet1.DOCX]

Supplementary Material

# Supplementary Data – Appendix of Experimental Advances

# Endometrial Organoids – Generation Techniques

Turco et al. (2017) cultivated endometrial and decidual tissue isolates with epithelial cells within Matrigel droplets and basal medium supplemented with epidermal growth factor (EGF), hepatocyte growth factor (HGF), fibroblast growth factor (FGF10), Noggin, R-spondin-1, A83-01, and nicotinamide, which had the most crucial effect on the quality of organoids. The organoid derivation efficiency in the case of decidual tissue was 96%, and of non-pregnant secretory endometrium, 100%. Organoids strongly expressed glandular epithelial markers such as *MUC1, E-CADHERIN, CK7,* and *EPCAM.* Gene ontology analysis showed the presence of epithelial markers (CDH1, CLDN10, and EPCAM), markers of mucosal secretory cells (PAX8, MUC1), and markers of glandular secretion in general (PEAP, KLK11, and MUC20) in both organoid types.

Boretto et al. (2017) cultivated glandular-type fragments from mouse endometrium in a medium containing a cocktail of growth signaling factors, including Wingless and Int1 (WNT) activators (WNT3A; R-spondin 1), epithelial cell mitogens (EGF, FGF10), Noggin, and A83-01. The medium was further supplemented with insulin-transferrin-selenium (ITS) because of its mitogenic effect on endometrial epithelium.

Regarding the endometrial organoids from human endometrial biopsies, the basal medium was similar to the previous one used for generating mouse organoids with N-acetyl-L-cysteine and SB202190, compounds generally applied in human organoid formation (Sato et al., 2011). Contrary to the mouse endometrial organoids, it was not necessary for human ones to use WNT3A for their further expansion; however, RSPO1 was needed for their adequate long-term expansion.

Haider et al. (2019) used as the primary cell population the human decidual cells and stromal cells. After their isolation, cells were embedded in Matrigel domes, and the basic organoid medium used for cell cultivation was supplemented with CHIR99021 (glycogen synthase kinase 3b inhibitor) and the same factors as those mentioned in previous studies, specifically Noggin, A83-01, R-spondin, and EGF. It was found that WNT pathway activators (R-spondin and CHIR99021) had the most crucial effect on efficient organoid formation. Subsequently, endometrial organoids started to appear after 7 to 10 days, displaying 100% derivation efficiency. Further immunostaining and PAS staining demonstrated the secretory abilities of organoids containing glandular epithelium. These precisely prepared organoids were treated with ES for 10 to 14 days to mimic the proliferative phase of the endometrial cycle. Surprisingly, the cilia marker ac-TUB showed the presence of multiple motile cilia only on epithelial cells of ES-treated organoids compared to the control group recapitulating ciliogenesis of the late proliferative phase *in vivo*.

Abbas et al. (2020) created a multicellular endometrial organoid model from endometrial biopsies and decidual stromal cells. The major difference compared to previous protocols was represented by the seeding of stromal cells and endometrial organoids on the scaffolds. In contrast, endometrial and decidual stromal cells produced ECM rich in collagen type I, II, III, IV, and V. TEM revealed microvilli on apical surfaces of epithelial cells and fibrous material underneath. Additionally, the formation of tight junctions and the presence of glycocalyx and lipid droplets were detected.

Jamaluddin et al. (2022) came up with an alternative to artificial ECM in form of decellularized endometrium. Decellularized ECM was enriched by selected proteins involving soluble and insoluble collagen forms with increased hydroxyproline concentration and sulfated glycosaminoglycans. Moreover, according to proteomic and microspectroscopic analysis, organoids were comparable to native endometrial tissue. As a next step, the authors decided to generate ECM hydrogel-derived porous scaffolds using an ice templating technique for better organoid cultivation. Seeded endometrial cancer cells and primary human endometrial cells were actively dividing to form 3D structures resembling organoids, demonstrating the ECM hydrogel-based scaffolds' promising potential.

Gnecco et al. (2023) co-cultured donor-matched endometrial epithelial organoids and endometrial stromal cell populations in engineered ECM for up to 15 days. Subsequent immunostaining revealed adequate morphology of both cell types, while cells were viable with minimal cell deaths. Besides that, the results of multiple analyses demonstrated the ability of this model to recapitulate distinct hormone-induced changes similar to a 28-day menstrual cycle.

# Endometrial Assembloids – Generation Techniques

Rawlings et al. (2021a) first generated gland-like organoids by cultivating Matrigel-seeded endometrial epithelial cells in an expansion medium according to the abovementioned Turco et al. (2017) protocol. The cells were further treated with progestin and cyclic AMP analog, providing adequate secretory differentiation of the gland-like organoid. The addition of endometrial stromal cells subsequently modified this organoid. Single-cell suspension of stromal cells combined with the epithelial cells were seeded in a hydrogel containing 97% type I collagen and 3% type III collagen, imitating the ECM composition of secretory endometrium in the mid-luteal phase of the ovarian cycle. These epithelial-stromal assembloids were further decidualized with 8-bromo-cAMP and progestin for four days. Immunofluorescence and single-cell transcriptomics revealed that the generated decidualized assembloid closely mimicked the *in vivo* endometrium in the given phase.

Shibata et al. (2024) generated an epithelial structure called apical-out endometrial organoid (AO-EMO), which was later combined with stromal cells (SCs), as well as self-formed endothelial elements. Only after these organoids were combined with human embryonic stem cell-derived blastoids the authors used the term assembloid. Nevertheless, the organoid was generated in a medium supplemented with EGF, CHIR99021, SB202190, and Y27632. After that, the organoid, human endometrial stromal cells (ESCs), and human umbilical vein endothelial cells (HUVECs) were introduced into a collagen-based gel, producing a composite structure termed AO-EMO + eSC/HUVEC. Next, AO-EMO + ESC (with or without HUVEC) were cocultured with blastoids in a floating culture. The culture medium included EGF, FGF for growth promotion, and LIF, which were supposedly involved in implantation. From Day 2, IGF-1 was added to promote inner cell mass proliferation, and BMP-4 was added to encourage trophoblast development. Only this final product was termed the feto-maternal assembloid.

Tian et al. (2023) created EnAOs by combining epithelial and stromal cells in ice-cold ECM of three types, Matrigel, Collagen I, and a combination of both (MAC), to determine which provides the best ECM properties. MAC was found to most closely resemble the ECM of the endometrium *in vivo*. To generate the ALI-EnAOs, EnAOs were combined with MAC in a 1:20 ratio and injected into the upper chamber of the Transwell system, which consists of a permeable membrane that separates two compartments within a culture dish and is used for studying processes such as cell migration, invasion, and adhesion. Glandular structures from the same donor were then added into the upper chamber, while the chamber below was filled with expansion medium, and the liquid from the upper chamber was removed. Assembloids generated by the mentioned ALI method displayed better cell composition, anatomical structure, and gene expression patterns, concretely the upregulation of *WOI* genes, *SPP1* gene, *GPX3* gene, *DFB1* gene, and *ARG2* gene.

# Trophoblast Organoids and Blastoids – Generation Techniques

Turco et al. (2018) derived trophoblast organoids from human placental tissue. Initially, first-semester chorionic villi were digested with trypsin-250 and collagenase V. Digests were then pooled, washed, and re-suspended in Matrigel on ice and plated in a culture plate overlaid with Trophoblast Organoid Medium containing advanced DMEM/F12, N2 supplement, B27 supplement minus vitamin A, primocin, N-Acetyl-L-cysteine, L-glutamine, ALK-4, -5, -7 inhibitor, A83-01, CHIR99201, recombinant EGF, Rspondin-1, FGF2 and HGF, Y-278632 and PGE2. By this technique, the authors generated a complex 3D structure resembling the placenta in the form of an extravillous trophoblast.

Fan et al. (2021) used iPSCs to generate blastoids instead of embryonic stem cells. First, iPSCs were generated by electroporating fibroblasts with episomal vectors. iPSCs were cultured in a human ESC medium, digested into single cells, and seeded. After 12 hours, the human ESC medium was replaced by a human N2B27-LCDM medium. Dome-shaped EPS colonies formed after 3-6 days. Subsequently, EPSs were used to generate EPS-blastoids. EPSs were placed in a plate pretreated with Matrigel in a BMP4 differentiation medium, which yielded trophoectoderm (trophoblast) cells (TEs). Then, TEs were mixed with EPSs and cultured in a medium containing beta-estradiol, progesterone, and N-acetyl-L-cyseine for six days. EPS-blastoid was formed on Day 5.
